# Experiences of American Health Departments, Health Systems, and Community Organizations in COVID-19 Vaccine Provision for Refugee, Immigrant, and Migrant Communities

**DOI:** 10.4269/ajtmh.23-0034

**Published:** 2023-07-10

**Authors:** Christine M. Thomas, Katherine Yun, Nadège U. Mudenge, Seja Abudiab, Diego de Acosta, Windy M. Fredkove, Yesenia Garcia, Sarah J. Hoffman, Sayyeda Karim, Erin Mann, M. Kumi Smith, Kimberly Yu, Elizabeth Dawson-Hahn

**Affiliations:** ^1^ Division of Infectious Diseases and International Medicine, Department of Medicine, University of Minnesota, Minneapolis, Minnesota;; ^2^ Perelman School of Medicine, University of Pennsylvania, Philadelphia, Pennsylvania;; ^3^ Children’s Hospital of Philadelphia, Philadelphia, Pennsylvania;; ^4^ Community Leadership Board, National Resource Center for Refugees, Immigrants, and Migrants, Minneapolis, Minnesota;; ^5^ Department of Pediatrics, University of Washington, Seattle, Washington;; ^6^ National Resource Center for Refugees, Immigrants, and Migrants, Minneapolis, Minnesota;; ^7^ Center for Global Health and Social Responsibility, University of Minnesota, Minneapolis, Minnesota;; ^8^ Seattle Children’s Research Institute, Seattle, Washington;; ^9^ School of Nursing, University of Minnesota, Minneapolis, Minnesota;; ^10^ Division of Epidemiology and Community Health, School of Public Health, University of Minnesota, Minneapolis, Minnesota

## Abstract

Effective provision of COVID-19 vaccines could mitigate the disproportionate impact of the COVID-19 pandemic experienced by many immigrant communities. To describe organizational experiences in using COVID-19 vaccination programs, qualitative interviews were conducted from September 2020 to April 2021 with representatives from public health, health system, and community organizations responding to the COVID-19 pandemic among immigrant communities across the United States. Interviews followed a semistructured interview guide and were audio recorded, transcribed, and coded. A latent thematic analysis was facilitated by Dedoose software. Interviews representative of 18 public health departments, 20 healthcare systems, and 18 community organizations were included in the analysis. Five identified themes referenced the importance of 1) appreciating community and individual heterogeneity in health priorities and attitudes; 2) addressing vaccine fears with trustworthy messages; 3) ensuring equitable access to vaccine opportunities; 4) making substantive investments in community partnerships and outreach; and 5) adapting to meet new needs. It is essential that vaccine efforts consider community heterogeneity, communicate in a trustworthy and culturally and linguistically appropriate manner, strive for equitable provision of care, build partnerships, and learn from prior experiences.

## INTRODUCTION

The COVID-19 pandemic has disproportionately impacted refugees, asylum seekers, immigrants, and migrants (hereafter referred to collectively as “immigrants”).^1^^,^^2^ The reasons vary by individual and community but can stem from factors such as employment in essential sectors, chronic illness, reduced access to health care, stigma and systemic racism, and challenges advocating for workplace protections as a result of language barriers or tenuous immigration status.^1^^–^^10^ Because COVID-19 vaccines may mitigate this impact, ensuring equitable access to vaccines for immigrant communities is essential.^2^^,^^11^ Unfortunately, many immigrant communities experience barriers to vaccination, including language and literacy barriers, inequitable access to health care, knowledge gaps, vaccine hesitancy regarding newer vaccines or differing beliefs about disease, or mistrust in government.^12^^–^^15^ Effective provision of COVID-19 vaccines to reduce the pandemic’s impact among these communities necessitates addressing these barriers.

In the United States, COVID-19 vaccine provision has relied heavily on public health agencies to coordinate allocation and supply. With increasing availability of vaccines, health systems have become predominant providers of vaccines. Community organizations have played an important role in outreach and developing innovative methods to support vaccine uptake. As such, each of these organizational types is well suited to provide expert insight about vaccination programs with immigrant communities that may be helpful for ongoing and future public health campaigns. In this study, we describe the triad of experiences of public health departments, health systems, and community organizations working to ameliorate the impact of the pandemic on immigrant communities through COVID-19 vaccination programs.

## MATERIALS AND METHODS

This project was funded by the National Resource Center for Refugees, Immigrants, and Migrants (NRC-RIM) to better understand the needs of health departments and community organizations working to ameliorate the impact of the pandemic on immigrant communities and to identify promising practices for dissemination to other organizations. The project was deemed nonhuman subject research by the University of Minnesota and exempt by the University of Washington. The primary focus of the parent project, which started in September 2020, was case investigation and contact tracing (CICT). The focus of this specific analysis was vaccination.

For the parent project, we conducted semistructured individual or group interviews from September 2020 through April 2021 with a purposive sample of informants from 60 public health departments, healthcare systems, and immigrant-focused community organizations throughout the United States. For this vaccine-specific analysis, we included 56 interviews in which COVID-19 vaccines were discussed (Table 1). The sample included organizations engaged in and/or knowledgeable about CICT within immigrant communities across the 10 U.S. Department of Health and Human Services regions,^16^ with efforts to include those located in rural and nonrural areas, local organizations, those with a statewide focus, and those working with different immigrant communities (e.g., migrant farmworkers and refugees) from a wide range of language groups (e.g., Spanish, Arabic, Nepali, Swahili, and others). We recruited organizations via English-language e-mails and webforms shared with existing networks, including the Association of Refugee Health Coordinators, National Association of County and City Health Officials, Society of Refugee Healthcare Providers, Migrant Clinicians Network, American Academy of Pediatrics Council on Immigrant Child and Family Health, International Rescue Community, and the Community Leadership Board of the NRC-RIM.

**Table 1:** Characteristics of participating organizations

Organizational variable	Public health department	Healthcare system	Community organization	Total
	(*N* = 18)	(*N* = 20)	(*N* = 18)	(*N* = 56)
Total number of interviewees*	29	20	21	70
Location, by HHS region†				
1 or 2	1	3	3	7
3 or 4	7	6	5	18
5 or 6	4	3	1	8
7 or 8	2	1	1	4
9 or 10	4	7	8	19
Organizational level‡				
Local (city/county)	7	20	15	42
State	11	0	1	12
Regional	0	0	2	2
Immigrant-specific organization§	10	4	18	32
Populations served‖				
Refugees	8	14	9	31
Migrant workers	8	2	8	18
Other immigrants	9	16	6	31
Interviews completed after COVID-19 vaccine EUA¶	5	10	10	25

EUA = emergency use authorization; FQHC = federally qualified health center; HHS = U.S. Department of Health and Human Services.

* Some organizations requested group interviews with two or more staff members.

† HHS region where the organization was located (https://www.hhs.gov/about/agencies/iea/regional-offices/index.html).

‡ Organization level was categorized as local (e.g., city or county) even if it was part of a statewide, regional, or national group if the operational unit that participated in the interview was focused on a local area. For example, an interview focusing on FQHC city-wide programming would be categorized as “local” even if the FQHC was part of a statewide FQHC network.

§ We categorized organizations as immigrant specific if the organization as a whole or the operational unit within the organization (e.g., a state refugee health program within a department of public health) focuses specifically on immigrant communities.

‖ Many organizations work with more than one population.

¶ Defined as an interview completed after the first EUA for a COVID-19 vaccine on December 11, 2020.

Each interview followed a semistructured interview guide that included exploratory questions about vaccination. The wording of each question was developed by the project team and updated after the U.S. Food and Drug Administration issued emergency use authorizations (EUAs) for vaccinations against COVID-19 (e.g., “[Assuming/Now that] a vaccine is found to be safe and effective, what are the most important actions or activities to ensure adequate vaccination coverage among refugee, immigrant, and migrant patients in your health system/health center?”) (Supplemental Table 1). We followed the thematic analysis approach described by Braun and Clarke.^17^ Interviews were audio recorded, professionally transcribed, and deidentified. A three-person coding team met weekly to develop three preliminary code books (public health, healthcare system, and community). Two members of the coding team coded the first five interviews in each group using Dedoose (Los Angeles, CA)^18^ to ensure consistency and to finalize the codebooks, each of which included codes pertaining to vaccination programs. One coding team member coded the remaining interviews. After all the interviews were coded, a two-person analysis team began collating the codes into thematic patterns using a semantic approach, considering the data at an explicit level.^17^ The themes were then refined, named, and defined. Key illustrative excerpts from the themes were extracted.^17^

## RESULTS

Five themes were identified from interviews representative of 18 public health departments, 20 healthcare systems, and 18 community organizations, which spanned the time preceding the EUAs for COVID-19 vaccines through the early phases of vaccine rollout among the general public. Here we present each theme followed by a description that includes supporting quotes and context for the selected in vivo theme titles (Table 2).

**Table 2:** Supporting quotes for themes

Theme 1: “In terms of vaccines, the different reactions I get are from alpha to the omega.”
“In terms of vaccines, the different reactions that I get are from alpha to the omega, I mean A to Z, from absolutely I need this vaccine—and those people are generally interestingly often not highly educated, often people who certainly—most of the [immigrant community] that I would relate to would say when can we have this vaccine? Very receptive to vaccines in general. And then in the more—some more possibly anti-government, I would say, populations they’re going to say, ‘Heck no.’ Well, they’d use other language, but they’d say no, they’re not going to have that—they don’t want to use that vaccine and they’re not going to get it and they’re never going to get it.” —Public Health Department
“When I get that information I can send it to you, but what we’ve heard is (and, sorry, these are informal surveys; we created a short survey sheet and just would ask patients) there are many people that were really distrusting of the vaccine. There are lots of conspiracy theories, like Obama created the vaccine to control people, and just crazy things. Then there’s a lot of like, ‘No one knows whether it’s effective,’ a lot of fear of side effects. It really depended on the community. Lots of different communities believe different things, but there really was a large reluctance to get this vaccine, and so it’s definitely less than 50% willing to be vaccinated, which I think as a general population we’re like 70% to 80%, so it’s a lot less than the general population.” —Health System
“We have a group that, obviously, is very interested in the vaccine and we have other groups that are very skeptical and think that it’s engineered to just get more people sick. And we just started, not too long ago, obviously, with education on the vaccine and this is why tomorrow, getting doctors door knocking, we put together this strategy, because we know that we need to be more out there and talking to people about vaccination.” —Community Organization
“We have two groups; we’re kind of divided. We have a lot of community members who are excited about the vaccine and they just can’t wait for their turn. We have a good number of community members who took it already…. There’s the other group, where they’re like, ‘you know, no, this is bad for us.’ ” —Community Organization
“Some of them are OK with it because, again, vaccination…. Back home some people don’t have access to vaccinations, so when they receive free vaccines they take it because they’re told it’s supposed to cure X disease or whatever. Some are OK with it; others aren’t because of history and when it comes to vaccination and black folks and [immigrant community] folks being used as guinea pigs to test vaccines on them and sterilize women, so you have those two camps when it comes to the community.” —Community Organization
“So one group may have doctors and dentists that are—already understand the basics and are asking about how the vaccine is actually made to folks that don’t even know what their immune system is.” —Public Health Department
“Yeah, people are really praying that they should have the vaccine because they are really mentally not feeling safe when they are outside. I think that everyone wants to have safety, to have the vaccine to make sure that they are not receiving this COVID-19.” —Community Organization
Theme 2: “Like I said, we tackle the vaccine fear.”
“So much has been going on in my community. We did little surveys, about 80% or 90% of them at a church; we did take a survey. They said they’re not going to take it or they’re going to wait. They talk about all of what I just said. We’ve been doing some information sharing and all that and education. We’ve been slowing down a little bit and now we’ve just started full speed, doing education, sharing information. Like I said, we tackle the vaccine fear. What is a vaccine? A lot of people don’t even know what a vaccine is. We start with that topic, building from that, and in the process why the vaccine is happening so fast. Technology played a big role in the rapidity of the vaccine.... There’s so much to it, so people feel like it would have to be medical scientists that should be in that conversation.” —Community Organization
“I think we’ve got to be really transparent about what we know and what we don’t know, sharing—being willing to share all the information that we have available with it. I think that’s going to be across the board. I don’t think that’s specific to [immigrant] communities. That’s a lot of our communities in [state]. Everybody wants the information, but I think too, we need to figure out ways to make space to listen to what the concerns are and recognize that that takes a lot more time than most of our clinicians have in the course of a typical visit. Then how are people accessing that information if they can’t ask their trusted clinician? Who’s giving them information? How do we make sure that public health is there saying, ‘Hey, this is—I’d be happy to hear more about that. Tell me more about that. Let me give you this information’ or whatever else it is. Again, going back to those trusted messengers as well and making sure that they have the right information. I think too, we’ve got to be careful. The information will change over time again. I think every time that happens, if we don’t own it to some extent, then we lose trust in the process.” —Public Health Department
Theme 3: “It’s not equal access to care for everybody like we wish.”
“Unfortunately, we have a very broken system. It’s not fair. It’s not equal access to care for everybody like we wish we could do. Perhaps there are some communities in the country that are different. I don’t know. But in our community, for instance, right now, I see that it’s like this black market that is grabbing onto some of the vaccines and giving it to the people to get favors of politicians, or the politician giving some just so they can get popular with their constituency and giving it to certain groups…. So you can see the disparity right there and then, that there is no system. They put everything on the website in English with a period of two hours to [register]. Our people don’t speak English. Our people don’t have a computer. Our older people that were targeted for them at the first, they don’t even know how to turn on a computer. They could do maybe Facebook, they maybe could do texting, but not that system that they established. Totally insensitive, again, to the population.” —Community Organization
“It’s distrust in the federal government in general and motivations—that’s it; it’s definitely not the mechanism of the vaccine or anything—which is layered on baseline vaccine hesitancy, which is slightly higher than the general population, particularly in our [immigrant] communities, and so it’s compounding, in that sense.” —Health System
“But the types of issues that come up for immigrants are the same ones always: is it going to be bilingual hotlines that people can call in and get the vaccine even if they don’t have a doctor, etc. We just had a conversation this morning because we had set up a hotline with the [center] for the testing, and we’ll probably adapt it to that. But the issue about whether people will be required to show any form of ID, because I think that, while many people do have an ID, some people don’t. They don’t even have a passport. They don’t have anything, or if they have an ID, maybe from, like, people move around for work, so hopefully there won’t be strong ID requirements. I don’t think Social Security is going to be required, but I don’t see why they would require that, and I haven’t heard that that would be an issue.” —Public Health Department
“I think the other thing around COVID vaccines is just what we’re doing now, and my colleague, who has the patient navigators and wants to do more funding, is actively outreaching patients on the phone, and that gets tricky. I don’t know what our response rate is ultimately going to be, but providing real information, and saying it looks like you or your child is behind on this vaccine, we’d like you to get an appointment, can I help arrange to get an appointment, you don’t have insurance, let me get you into our enrollment. We have hired staff within our hospital system that specifically link people and try to identify health insurance, and if they can’t qualify for anything, CHIP [Children’s Health Insurance Program] or otherwise, then they would get on our sliding scale. So it’s getting all this information out, and addressing all the barriers so that the only reason, honestly—transportation as well—the only reason that they would refuse is fear, they don’t believe in vaccines, the usual stuff. But let’s take all the rest of the barriers out and get them in.” —Health System
“And we actually take a lot of care with our patients, so we make sure that mostly it’s all eligible patients; we’re only doing patients, mainly, now, and getting through the elderly, the medical conditions, and the work groups. And it’s been really hard because, in many places, you see people go online and be able to schedule; our communities can’t do that, even when we created one, because of the digital divide. So our staff are basically calling, and we also set up a helpline that they can call, in different languages. We have staff who speak different languages that can assist them in getting the appointment. That was really major resources, both in terms of the vaccine clinic itself but also the outreach, the intense outreach that we had to do.” —Health System
“But then in another few weeks, technically, everybody should qualify for getting the vaccine, and then we are planning on vaccinating, like doing the mass immunization specific to our communities, because it is so challenging for everybody from the community to go get vaccinated in the doctor’s office or in the hospital setting, so we are trying to bring the vaccine to our own communities that way and then explain then what the benefits are and how the vaccine prevents them. So hopefully we’ll be able to vaccinate people pretty soon, and I think when they speak to the state as an agency or as a representative in each and every state, they would get the priority because of the minority issue, because of the language barrier issue, it is so hard for each and every office of the hospital to administer the vaccine. So if any community organization comes up with the idea and then plans to administer the vaccine, I think the state is willing to work with them. I wish there would be an organization in each and every state that worked that way and then vaccinated our people, you know?” —Community Organization
“I think we have to take the vaccine to those communities [without access to a regular clinic or services]. We can’t just…rely on them to go to a clinic or something. I think you’ve got to go there. And so I’ve been beating the drum about that…. The congregate setting, you know, folks are living in a congregate setting, you’ve got to take the vaccine to them. Let’s go. Let’s go, let’s go.” —Public Health Department
Theme 4: “It’s going to just take overinvestment in community outreach and partnerships.”
“It just has to be intentionality. You probably saw the National Academy of Sciences and Medicine recommended prioritizing I think they said minority groups, so basically people who have been disproportionately affected by health disparities. Obviously, it would be difficult to profile in that sense, but it’s not difficult to overinvest in reaching hard-to-reach communities. And it’s going to be especially difficult, because whereas we can do community-based vaccination efforts for flu, we can’t do that with the Pfizer and Moderna vaccines because of their ultracold storage. Those have to be delivered at clinics that have these megafreezers, and so that’s going to be a real challenge until we get the AstraZeneca that’s a more stable vaccine out there into the communities. So yeah, it’s going to be a challenge, but it’s going to just take overinvestment in community outreach and partnerships.” —Health System
“I think those partnerships are going to be more important than ever in terms of who gets prioritized and how we roll out a vaccination, how we make it available, and then what the uptake looks like, and monitoring the uptake.” —Public Health Department
“I don’t know. To tell you the truth, they’re [public health] a little more restrained, keep to themselves. It’s not the right way. I know other counties, they’re more open. They want to collaborate with other community organizations and all that, even though I’m part of a bigger [COVID-19 taskforce]. The [local governmental official] is part of it and then you have a lot of other nonprofits being part of it. They’re there, but at the same time it feels like they’re not. Sometimes they are very defensive about what they do. It’s one person—I don’t know if it’s that person’s attitude. It may not be the whole organization itself, but we feel like there’s a lack of integration, lack of openness for partnership or collaboration.” —Community Organization
“The purpose of these local community organizations is to have trust relationships with the communities. [One morning each week vaccines are provided], but sometimes it’s chaotic because people don’t show. You have a waiting list and you open a bottle for the vaccines. I have to run and make phone calls. ‘Vente ahorita! Run and come in right now,’ [community] style. Yes, the first [day] there were [more than a hundred] and the person in charge for that day for the vaccination site, she said this is unbelievable and historical. Compared with the rest of the vaccination sites, we didn’t throw away any single vaccine.” —Community Organization
“I have to say I’m really happy with the way the department of public health has helped us. I’m going to talk about vaccines and even right now they’ve offered, ‘if we block out a time just for the [immigrant] communities on our time slots, can you guys come out and have them sign up for those vaccine slots?’ So just having the trust and having them see the work that you do, I think that’s really important. [Interviewer: Can you talk a little bit about the partnership and what it looks like and what’s happening?] Of course right now a lot of the events we’re geared towards is the vaccine. Like I said, we still hold COVID testing events. The [academic institution] mobile van is still going out for COVID testing, but right now they’ve also added the vaccine events. Like on Thursday mornings, even with [organization] at first it was just COVID testing, but then as the vaccines started to roll out, they were like, ‘we can bring out 50 vaccines along with COVID testing. So if you have 50 people for us, we can do it every week.’ So that’s what we’ve been doing every week with [academic institution] also. Also, if [doctor] has some extra in the afternoon while we’re holding our afternoon testing out front, he’ll run out front, ‘may I have this many people?’ So of course we do follow the tiers for those that are eligible for those vaccines. We always have a waitlist available on hand at [community organization] and then we’ll just call off from that list. [Interviewer: So you’re doing at the same time, same event, mobile testing and mobile vaccination.] Yes. [Interviewer: How has that gone?] It’s gone great. I also want to say that the [state] also gave [organization] two events for vaccines for just farmers, because here in [city] there’s a lot of [immigrant] farmers…we were vaccinating like 300 farmers. I really appreciate how [they] picked our organization. ‘We’re going to give you 300 vaccines; can you find 300 farmers?’ We’re like, yeah, definitely. We did that. We did 300 the first time and then the second time that [they] gave us a second date we did 100, so we did a total of about 400 farmers.” —Community Organization
Theme 5: “We’re doing a lot of pivoting when there’s need.”
“It’s all of us going out into the community with our mobile clinics or outreach, or walking and providing services to our patients at their homes because of COVID. We’re doing a lot of pivoting when there’s need, and doing a lot of innovative new things to reach our communities, which has been hard for a lot of families.” —Health System
“I’m feeling that because everything is so rushed, this is so new, I’m afraid, and I say this in all the spaces I’ve joined for the vaccine is, what did we learn about the COVID testing that we can learn from the vaccine experience? If we just do a bunch of open pop-ups, come and get your vaccine, it’s not as simple as that. It really needs to be targeted and started earlier, the education on the vaccine, because a lot of people still fear it; they don’t want to get it. There’s some really good lessons learned from the COVID testing that could be used for the vaccine.” —Community Organization
“It took three months of pushing and pushing and pushing and working with the county and getting it before our system really got behind it. And the partnership we have now with [local public health] is very, very strong. They said they want us to be their clinical partner for everything, so now we’re partnering with them doing vaccines, and that has been really fun.” —Health System
“Just at the time when we think we have a process in play because we have it all down pat, now we’ve added on this last two weeks new testing sites and new people and what does that look like for processes. And now we’re trying to plan for vaccinations that we don’t know a whole heck of a lot about.” —Public Health Department
“Now the conversation we’re having is what are the implications for COVID vaccines and at the last meeting, which was just this past weekend, was when we really began to have in-depth conversation about okay, where do we go, how do we approach this. Part of the challenge is, first of all, we didn’t even have any clear directive or guidance as to when the vaccines were going to be ready, how it was going to be rolled out, what we were going to have to do…. Now that we’re beginning to get the answers at this last meeting that we had on Sunday, we’re now talking about okay, how can we do the messaging piece and how can we get community buy-in.” —Public Health Department
“It doesn’t feel like scarcity, like it did last year, where it was just like, oh, taking everything you’ve got to just tread water. And there was so much pressure on the local resources, and now there’s a whole lot more funding, and there’s just a ton of information and resources available. However, the public health folks are exhausted. Everybody’s exhausted. It’s been a year, and people are tired, and now we’re trying to get vaccine out as fast as possible.” —Public Health Department

### Theme 1: “In terms of vaccines, the different reactions I get are from alpha to the omega.”

Participants across organizations discussed the heterogeneity of attitudes about COVID-19 vaccines among communities and individuals, with one employee of a public health department describing a continuum of vaccine reactions “from alpha to the omega.” At a clinic serving a high percentage of refugees and immigrants, it was noted that “lots of different communities believe different things.” A participant representing an organization primarily serving immigrants noted, “We have a group that, obviously, is very interested in the vaccine and we have other groups that are very skeptical….” Another organization serving refugees similarly found, “We have two groups; we’re kind of divided. We have a lot of community members who are excited about the vaccine and they just can’t wait for their turn. We have a good number of community members who took it already.”

Reasons provided for differing reactions to vaccines related to historical experiences, health literacy, and risk perception. As a representative for a community organization working with immigrants and refugees from all over the world explained, “Back home some people don’t have access to vaccinations, so when they receive free vaccines they take it because they’re told it’s supposed to cure X disease or whatever. Some are OK with it; others aren’t because of history and when it comes to vaccination and black folks and [nationality] folks being used as guinea pigs to test vaccines on them….” Differences in health literacy were described by a public health department that hosted meetings with community leaders: “One group may have doctors and dentists that…are asking about how the vaccine is actually made to folks that don’t even know what their immune system is.” And the importance of risk perception was also shared by a participant: “People are really praying that they should have the vaccine because they are really mentally not feeling safe when they are outside. I think that everyone wants to have safety, to have the vaccine to make sure that they are not receiving this COVID-19.”

### Theme 2: “Like I said, we tackle the vaccine fear.”

**Figure 1.**
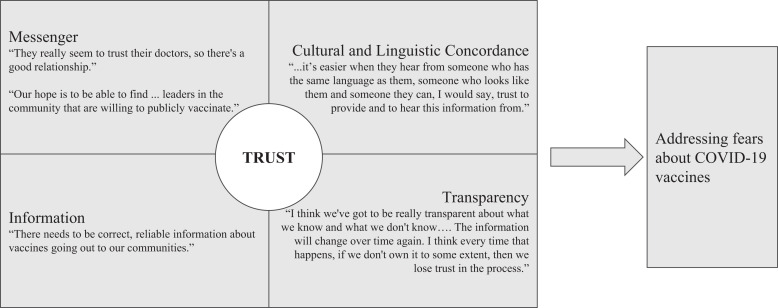
Key components to addressing vaccine fears.

Interviewees across all three groups emphasized the need for their organizations to partner with or serve as trusted, credible voices addressing community fears and misinformation about COVID-19 vaccines. The leader of a community organization serving migrant farmworkers described their work post-EUA as follows: “…now we’ve just started full speed, doing education, sharing information. Like I said, we tackle the vaccine fear. What is a vaccine? A lot of people don’t even know what a vaccine is. We start with that topic….” The leader of a public health program focusing on refugees described their planning process prior to the EUAs, saying, “Again, going back to those trusted messengers as well and making sure that they have the right information. I think too, we’ve got to be careful. The information will change over time again. I think every time that happens, if we don’t own it to some extent, then we lose trust in the process….” In addressing fears, interviewees described building a core foundation of trust, providing accurate information, prioritizing transparency, using culturally/linguistically concordant practices, and working with the right messengers, for example, physicians in some communities and community leaders in others (see Figure 1, with supporting quotes in Supplemental Table 2).

### Theme 3: “It’s not equal access to care for everybody like we wish.”

Challenges for communities in accessing vaccines were frequently mentioned. Many organizational representatives described the barriers faced by communities as “layered” and “compounding,” underscoring obstacles and inequities that preexisted and transcend the pandemic. As a public health department employee stated, “The types of issues that come up for immigrants are the same ones always.” These challenges include language and literacy differences, technological capability, concerns about immigration status and the government, limited access to health information and care, and concerns about cost. The leader of a community organization serving migrant farmworkers described the resulting inequity: “Unfortunately, we have a very broken system. It’s not fair. It’s not equal access to care for everybody like we wish we could do…. They put everything on the website in English with a period of two hours [to schedule a vaccine]. Our people don’t speak English. Our people don’t have a computer.”

Participants, especially health system providers, frequently discussed models their organizations intentionally implemented in an attempt to facilitate access to vaccination and circumvent these systems-level barriers.^19^^–^^22^ As one participant explained, “Let’s take all the rest of the barriers out and get them [immigrant community members] in.” Organizations circumvented barriers at multiple steps in the chain of events, leading to successful vaccination. To address the challenges of web-based and primarily English registration systems, culturally and linguistically concordant patient navigators participated in what was termed “intense outreach,” where they helped patients schedule appointments for vaccination over the phone in their preferred languages. Participants also emphasized the importance of ensuring appropriate interpreters were available at vaccination events and providing vaccines free of charge. Opportunities for vaccination were increased by extending the hours in which they were offered, utilizing innovative locations for administration (such as parking lots outside houses of worship, senior residential facilities or day centers, and mobile vans), and partnering with home care agencies already working in the community. Integral to overcoming barriers was a philosophy of bringing vaccination opportunities directly into the community. Participants from all three groups discussed this concept. A refugee community member working as a nurse noted “…because it is so challenging for everybody from the community to go get vaccinated in the doctor’s office or in the hospital setting, so we are trying to bring the vaccine to our own communities….” Another nurse at a federally qualified health center described the importance of going to communities, saying, “That way they don’t have to worry about access,” and a public health department employee emphasized, “You’ve got to take the vaccine to them. Let’s go. Let’s go. Let’s go.”

### Theme 4: “It’s going to just take overinvestment in community outreach and partnerships.”

Interorganizational partnerships were frequently discussed by interviewees, with their importance highlighted during each step of vaccine rollout. Prior to vaccine availability, a public health department employee noted, “Those partnerships are going to be more important than ever.” Unfortunately, partnership was not always well implemented and immigrant communities were not always prioritized in public health decision making. For example, the leader of an organization serving farmworkers described the local government’s COVID-19 task force as follows, “They’re there, but at the same time it feels like they’re not.”

In partnerships that were reported as effective, interviewees from all three groups described a pattern of linkage between public health, health system, *and* community organizations. This was especially evident in vaccine provision where public health departments provided vaccines, health system personnel administered vaccines, and communities advertised and organized vaccination opportunities. A particular advantage to partnering with community organizations was the ability to recruit and register community members for local vaccine events through word of mouth. When asked about strategies to avoid disparities in access to vaccination, one health system informant interviewed shortly prior to the EUAs noted that deep freeze requirements would make it more difficult to bring vaccines into community settings. To offset this barrier, they noted, “Yeah, it’s going to be a challenge. But it’s going to just take overinvestment in community outreach and partnerships.” This helped facilitate vaccine prioritization, ensuring allotted vaccines reached their intended communities, and allowed trusted messengers to build vaccine awareness within communities.

Often, these relationships predated the pandemic or, at the very least, predated the vaccine EUAs. In describing a program of this nature operating in early 2021 when the United States still had vaccine shortages, one community leader described their success as follows: “Yes, the first [day] there were [more than a hundred vaccines given] and the person in charge for that day for the vaccination site, she said ‘This is unbelievable and historical.’ Compared with the rest of the vaccination sites, we didn’t throw away any single vaccine.” Another community leader described the success of a similar program: “I have to say I’m really happy with the way the department of public health has helped us. I’m going to talk about vaccines and even right now they’ve offered, ‘if we block out a time just for the [immigrant] communities on our time slots, can you guys come out and have them sign up for those vaccine slots?’ …It’s gone great. …We did 300 the first time and then the second time that he gave us a second date we did 100, so we did a total of about 400 farmers.”

### Theme 5: “We’re doing a lot of pivoting when there’s need.”

**Figure 2.**
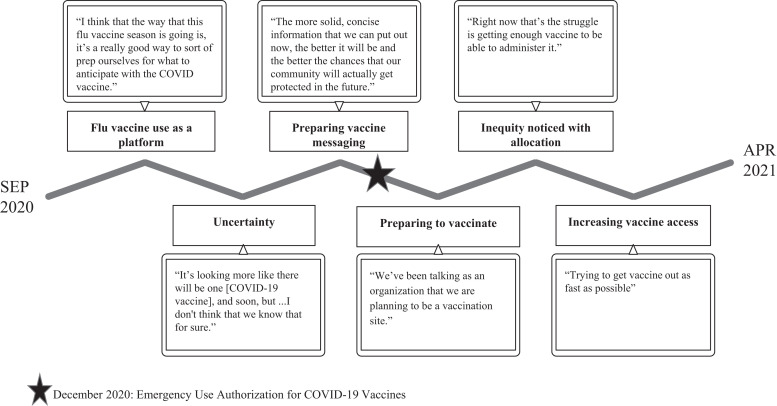
Conversations about COVID-19 vaccination changed over time.

COVID-19 vaccine rollout was characterized in part by how pandemic objectives changed as the response evolved. Over the course of interviews, conversations about vaccines changed. Earlier interviews included discussions about using influenza vaccination efforts as a springboard for a future COVID-19 vaccine and uncertainty about COVID-19 vaccines. Later interviews focused on preparing for and implementing vaccine rollout, frequently in the midst of inequitable allocation and access for immigrant communities (see Figure 2, with supporting quotes in Supplemental Table 2). During this period, participants also noted, “There’s some really good lessons learned from the COVID testing that could be used for the vaccine.” Participants shared that their organizations provided pop-up vaccine clinics in parks where testing events had been done, and expanded mobile van capability to include vaccination in addition to testing. Additionally, partnerships established during testing efforts were built upon during vaccine delivery. One health system provider discussed this dynamic with the county health department, saying, “They said they want us to be their clinical partner for everything, so now we’re partnering with them doing vaccines, and that has been really fun.”

The pivot to vaccines was also associated with implementation challenges related to a lag in information and guidance, and emotional exhaustion in those responding to the pandemic. A public health department employee explained, “Just at the time when we think we have a process [CICT] in play because we have it all down pat, …now we’re trying to plan for vaccinations that we don’t know a whole heck of a lot about.” Another elaborated, “Part of the challenge is, first of all, we didn’t even have any clear directive or guidance as to when the vaccines were going to be ready, how it was going to be rolled out, what we were going to have to do. We didn’t have those kinds of information….” In the setting of limited public health funding and human resources, one participant discussed the difficulty of finding energy to develop vaccination programs, saying, “…the public health folks are exhausted. Everybody’s exhausted.”

## DISCUSSION

Through interviews with representatives for over 50 public health departments, healthcare systems, and community organizations working to ameliorate the impact of the pandemic on immigrant communities, we identified five key themes for COVID-19 vaccination programs that may also have implications for other vaccination efforts, emergency preparedness, and community health as a whole. Application of these themes entails appreciating heterogeneity in health priorities and beliefs among communities and individuals, addressing vaccine fears with trustworthy messages, establishing equitable access to vaccine opportunities, ensuring that public health and health system investment in community partnership is substantive and sufficient to overcome barriers and prevent disparities, and adapting to meet new needs.

These findings are consistent with a larger body of research on vaccine access for immigrant communities. Pertaining to COVID-19 vaccines, heterogeneity in attitudes and beliefs pertaining to the vaccine,^3^ and the importance of trust in vaccine decision making,^23^^,^^24^ has also been identified through prior surveys and focus groups with immigrants. Similarly, advocates for immigrant health have called for equitable inclusion of immigrants in pandemic mitigation efforts through intentional partnership with communities, culturally appropriate materials, and language access.^1^^,^^2^^,^^12^^,^^13^^,^^25^^–^^28^

Although our findings are immediately applicable to COVID-19 vaccination efforts (including provision of booster doses and vaccinations for younger children) and will be helpful as new vaccines become available for other diseases, they also pertain to routinely provided immunizations, for example, annual influenza, scheduled childhood vaccine series, or age-appropriate pneumococcal (*Streptococcus pneumoniae*) and shingles (herpes zoster) immunizations. Preceding the COVID-19 pandemic, immigrants experienced lower rates of vaccination, with barriers stemming from knowledge gaps about vaccines and the diseases they prevent, insufficient healthcare access, and vaccine hesitancy among some communities toward specific vaccines.^14^^,^^29^ Intentional partnership and community engagement in vaccine campaigns coupled with the provision of culturally and linguistically appropriate information may mitigate some of the barriers related to knowledge gaps and vaccine hesitancy,^28^^,^^30^^,^^31^ whereas implementing innovative methods of vaccine delivery, such as pop-up or mobile clinics in the community, may help circumvent barriers stemming from insufficient healthcare access.^28^^,^^32^

It is essential that the lessons learned during this current pandemic be leveraged for future public health emergencies, and community health as a whole. During the H1N1 pandemic, focus groups with immigrants identified fear toward the vaccine, in part related to its novelty and the governmental or pharmaceutical companies’ role in development, and a lack of information about how to access the vaccine.^15^ Now, a decade later, we are again describing a need to address vaccine fears and provide vaccination opportunities to communities. The path to accomplish this relies upon sustained investment in communities, including partnerships that allow for nimble adaptation to emerging public health threats and rapid development of culturally and linguistically appropriate interventions that the community trusts.^33^^,^^34^ The time to build and sustain these partnerships is now, before, rather than during, the next pandemic.

A limitation of this project is that organizational representatives were invited to primarily share information about CICT rather than vaccine efforts. As a result, there are likely to be additional organizational practices and examples that we did not identify.

The findings from our interviews with representatives from public health, health system, and community organizations coalesced themes from COVID-19 vaccine efforts among immigrant communities that are helpful for other vaccine efforts, emergency preparedness, and community health. It is important for vaccine efforts to consider the heterogeneity of communities when developing interventions, address concerns in a culturally and linguistically appropriate manner that relies on and builds trust, strive for equitable vaccine provision, invest resources in communities and build on community strengths through partnerships, and learn from prior experiences to meet emerging health needs.

## Supplemental Material


Supplemental materials
