# Supplementary material for: Experiences of American Health Departments, Health Systems, and Community Organizations in COVID-19 Vaccine Provision for Refugee, Immigrant, and Migrant Communities
Source: Am J Trop Med Hyg. 2023 Jul 10;109(2):471–9. doi: 10.4269/ajtmh.23-0034 (PMC10397449; doi:10.4269/ajtmh.23-0034)
Supplement: Supplementary file 1 [file tpmd230034.SD1.pdf]

Supplemental Table 1. Semi-structured interview questions pertaining to COVID-19 vaccination

**Organization Type**	**Questions**
Public Health Department	Have you been involved in COVID-19 vaccination planning? If yes, in what ways?<br>Has there been any discussion [within the organization] of COVID-19 vaccination in RIM communities?
Healthcare System	Assuming a vaccine is found to be safe and effective [After the FDA EUA, this was changed to “Now that COVID-19 vaccines have been found to be safe and effective”], what are the most important actions or activities to ensure adequate vaccination coverage among RIM patients in your health system/health center?<br>What discussions, if any, have you/your health system/your health center had with RIM patients about a potential COVID vaccine?
Community Organization	What kinds of conversations, if any, have members of your community been having about COVID-19 vaccination?<br>What approaches might help community members understanding or willingness to receive this vaccine?<br>Do you have any other suggestions for how to improve uptake of a potential COVID vaccine in your community?

Supplemental Table 2. Supporting quotes for figures

Figure 1. Key components to addressing vaccine fears
Transparency:<br>"I think we've got to be really transparent about what we know and what we don't know, sharing – being willing to share all the information that we have available with it. I think that's going to be across the board. I don't think that's specific to [immigrant] communities. That's a lot of our communities in [state]. Everybody wants the information, but I think too, we need to figure out ways to make space to listen to what the concerns are and recognize that that takes a lot more time than most of our clinicians have in the course of a typical visit.<br><br>Then how are people accessing that information if they can't ask their trusted clinician? Who's giving them information? How do we make sure that public health is there saying, "Hey, this is – I'd be happy to hear more about that. Tell me more about that. Let me give you this information" or whatever else it is. Again, going back to those trusted messengers as well and making sure that they have the right information. I think too, we've got to be careful. The information will change over time again. I think every time that happens, if we don't own it to some extent, then we lose trust in the process." -*Public Health Department*
Messenger:<br>"They really seem to trust their doctors, so there's a good relationship. Most of the refugees have a really good relationship with their doctor, so, you know, if their doctor says, 'Yeah, it's safe and it's a really good idea to take it,' I think they'll take it. And then if [community organization] is backing that up with sort of a secondary source, so if we're saying, 'Yeah, we agree. It's good to take the vaccine,' then people will take it." -*Community Organization*
Messenger:<br>"And we've talked about this as a task force, as well, that there needs to be some sort of constant communication about these vaccines but in a way that actually makes sense for these communities. Our goal is really to try to influence people in leadership in these communities, especially in religious communities, and our hope is to be able to find, once it's more broadly available, leaders in the community that are willing to publicly vaccinate, and hope that that influences everyone else to get vaccinated." -*Health System*

Cultural and linguistic concordance:

"Some of the classes are educational sessions to answer people's questions or to provide updates… We just want to come and say, 'some of this information is not correct,' or, 'one thing that works for you might not work for everyone. Everyone's body is different. 'So just educate them and sometimes it's easier when they hear from someone who has the same language as them, someone who looks like them and someone they can, I would say, trust to provide and to hear this information from. That's very effective." -*Community Organization*

Information:

"I think this actually affects all populations, to be honest – perhaps even more minority because of the distrust of the medical research and the medical system that has happened – but I think we do have to address hesitancy. I'm hearing from others that even some health care workers have hesitancy. So it's not just the general public; it's also they, so I think there needs to be correct, reliable information about vaccines going out to our communities." -*Health System*

Figure 2. Conversations about COVID-19 vaccination changed over time

Flu vaccine use as a platform:

"I think that the way that this flu vaccine season is going is, it's a really good way to sort of prep ourselves for what to anticipate with the COVID vaccine. So, trying to be proactive in addressing the vaccine hesitancy and talking through side effects, efficacy, like, what does that actually mean." -*Health System*

Uncertainty:

"There may or may not be a vaccine soon or not soon. It's looking more like there will be one, and soon, but we—you know, I don't think that we know that for sure. There are committees that—if someone asks me about it, there are committees actively working on that through the county health system looking at equitable distribution of vaccines, and so, I would tell them that that's happening on a system-wide basis." -*Health System*

Preparing vaccine messaging:

"We had a meeting prior to that, that just kind of blanket – talk-talked about, um, communication in relation to the COVID vaccine because of course we feel like communication right now is when it needs to start to happen because people again are uncomfortable potentially with the idea of getting the vaccine. There's been a lot of misinformation surrounding the

vaccine, and so we feel like the more solid, concise information that we can put out now, um, the better it will be and the better the chances that our community will actually get protected in the future. So, um, yeah, we're absolutely having those discussions now and we're hoping that those partnerships with those agencies that are already well known to those communities will-will benefit us and will make them more comfortable getting that." *-Public Health Department*
Preparing to vaccinate:<br>"We've been talking as an organization that we are planning to be a vaccination site. We talked with the county a while back about becoming a POD, which is a point of distribution. I don't know if that's a common name or if it's just something [state] does, but they have open and closed PODs. An open POD would be available to anyone in the community, and you have to reach a certain percentage of the community. A closed POD, which is what we'll be, is you can vaccinate staff, patients, and their families. There's also another way through [state], so we're registered in both ways to get vaccine when it's available. We're making plans about how we will do that." *-Health System*
Allocation inequity:<br>"We're continuing to work with the health department. On our call yesterday they were telling us all the things they were doing and they said, what can we do to help. I said get us some vaccine. That's where we need the help. Right now that's the struggle is getting enough vaccine to be able to administer it." *-Health System*
Increasing vaccine access:<br>"It doesn't feel like scarcity, like it did last year, where it was just like, oh, taking everything you've got to just tread water. And there was so much pressure on the local resources, and now there's a whole lot more funding, and there's just a ton of information and resources available. However, the public health folks are exhausted. Everybody's exhausted. It's been a year, and people are tired, and now we're trying to get vaccine out as fast as possible." *-Public Health Department*
